# Supplementary material for: The 35th Anniversary of the Discovery of EPR Effect: A New Wave of Nanomedicines for Tumor-Targeted Drug Delivery—Personal Remarks and Future Prospects
Source: J Pers Med. 2021 Mar 22;11(3):229. doi: 10.3390/jpm11030229 (PMC8004895; doi:10.3390/jpm11030229)
Supplement: Supplementary file 1 [file jpm-11-00229-s001.pdf]

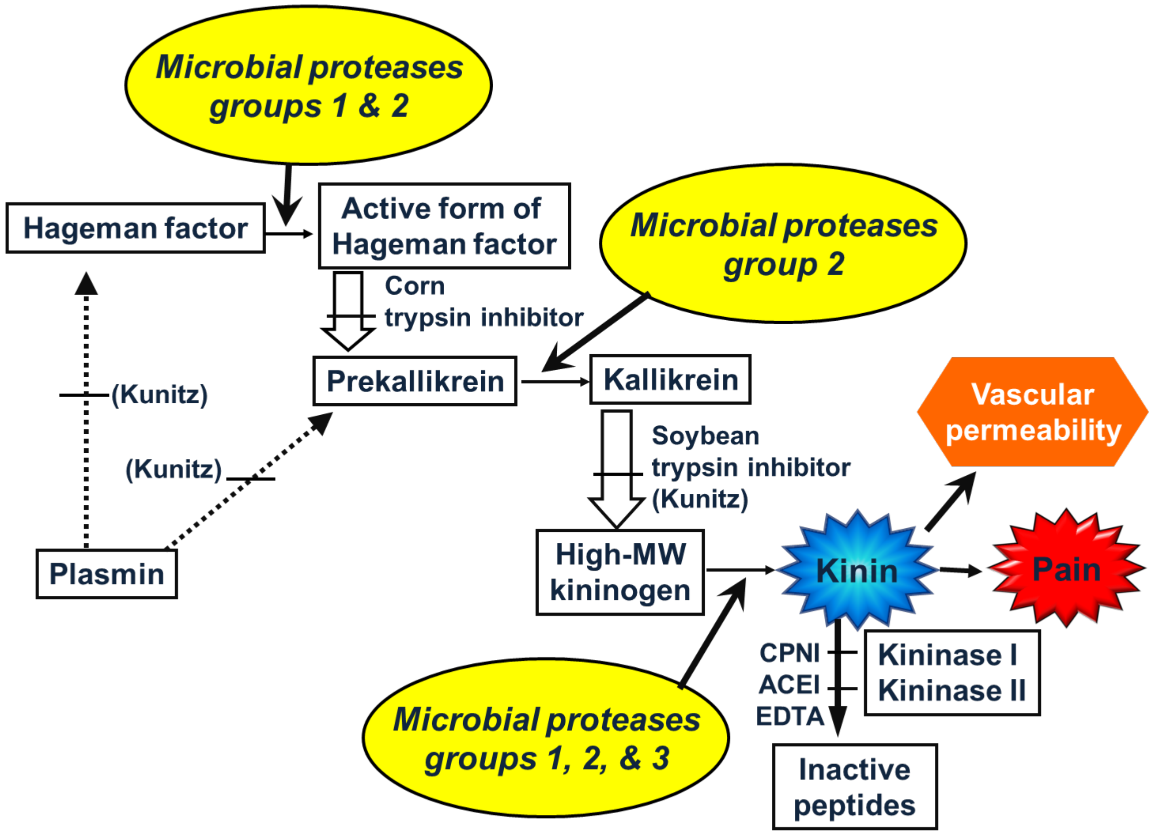

Supplementary Figure S1: The bradykinin (kinin)-generating cascade of host animals that is activated by various microbial proteases at different steps and inhibitors. All protease inhibitors in the plasma, including α_1-trypsin inhibitor and α_2-macroglobutin, cannot efficiently suppress these bacterial proteases. and α_2-macroglobutin is effective for only a short period. Similar to the protease cascade of kinin production that operates in parallel is the blood clotting (fibrin formation) cascade [4, 20-30, 34]. Kinin has multiple physiological roles, including pain induction, effects on vascular permeability, and effects on many inflammatory mediators (see Figure 1). CPNI, carboxypeptidase N inhibitor; ACEI, angiotensin-converting enzyme inhibitor; EDTA, ethylenediaminetetraacetic acid. (Adapted from [4,34].)
